# Engineering T cells for cancer therapy

**DOI:** 10.1038/sj.bjc.6602839

**Published:** 2005-10-25

**Authors:** W Mansoor, D E Gilham, F C Thistlethwaite, R E Hawkins

**Affiliations:** 1 Cancer Research UK, Department of Medical Oncology, University of Manchester, Paterson Institute for Cancer Research, Christie Hospital NHS Trust, Wilmslow Road, Withington, Manchester M20 4BX, UK

**Keywords:** chimeric immune receptor, antibody

## Abstract

It is generally accepted that the immune system plays an important role in controlling tumour development. However, the interplay between tumour and immune system is complex, as demonstrated by the fact that tumours can successfully establish and develop despite the presence of T cells in tumour. An improved understanding of how tumours evade T-cell surveillance, coupled with technical developments allowing the culture and manipulation of T cells, has driven the exploration of therapeutic strategies based on the adoptive transfer of tumour-specific T cells. The isolation, expansion and re-infusion of large numbers of tumour-specific T cells generated from tumour biopsies has been shown to be feasible. Indeed, impressive clinical responses have been documented in melanoma patients treated with these T cells. These studies and others demonstrate the potential of T cells for the adoptive therapy of cancer. However, the significant technical issues relating to the production of natural tumour-specific T cells suggest that the application of this approach is likely to be limited at the moment. With the advent of retroviral gene transfer technology, it has become possible to efficiently endow T cells with antigen-specific receptors. Using this strategy, it is potentially possible to generate large numbers of tumour reactive T cells rapidly. This review summarises the current gene therapy approaches in relation to the development of adoptive T-cell-based cancer treatments, as these methods now head towards testing in the clinical trial setting.

## 

The immune system has developed in order to protect against infection by pathogens and thereby prevent disease. With a greater understanding of immune cell function, there is now an increased awareness that the immune system actually plays a critical role in cancer prevention (Zou, 2005). Delineating this role of the immune system remains a key goal of basic research; however, the implication of these observations is that manipulating and boosting the power of the immune system may prove to be a potent cancer therapy (Murphy *et al*, 2005).

To this end, immune cells themselves are being increasingly investigated for potential cancer therapies, with a great deal of interest now focused on T cells in particular (Rosenberg *et al*, 2004). T cells are key effector cells of the adaptive immune system that perform critical activities, which includes target cell lyses, while working in conjunction with other immune cells to orchestrate the immune response through the timed production of cytokines (Shankaran *et al*, 2001). T cells respond to antigen as a result of the precise interaction of the T-cell receptor (TCR) with antigens presented on target cells. The antigen consists of peptides of varying lengths presented in the binding groove of molecules called the major histocompatibility complex (MHC). Critically, the type of T cell activated is dependent on the MHC presenting the peptide antigen. All nucleated cells express MHC class I proteins that can present antigens to CD8^+^ expressing T cells, while a more restricted sub-set of cells (generally called antigen-presenting cells (APCs)) express MHC class II, which specifically activate CD4^+^ T cells. The general defining features of these two sub-sets of T cells are that CD8^+^ T cells are considered to be the predominant cytotoxic cells (T_c_) while CD4^+^ T cells are thought to play a critical role in refining and optimising T cell and other immune cell responses and so are commonly referred to as helper T cells (T_h_). It is clear that these separations are simplistic and may not entirely reflect the *in vivo* functions of the T-cell lineages; however, these distinctions are relevant for a consideration of how T cells can be used for cancer therapy (Figure 1).

## ADOPTIVE T-CELL THERAPY: ALLOGENEIC T CELLS FOR HAEMOPOIETIC MALIGNANCIES

The power of adoptive T-cell therapy has been clearly demonstrated using donor lymphocyte infusions (DLI) for the treatment of a number of haematological malignancies (Kolb *et al*, 1995). Lymphocytes isolated from an allogeneic donor and given to a patient are thought to respond to tumour through MHC mismatches (either major or minor MHC mismatches) and subsequently eliminate tumour (the graft-*vs*-tumour effect). However, by the same process, they are also destructive to healthy host tissue. This unwanted side effect, graft-*vs-*host disease (GvHD), is associated with high rates of morbidity and mortality. This has limited the use of such therapy to a few specialist hospitals, where many strategies have been employed to control such toxicity (Goker *et al*, 2001). However, T cells are the critical component since their depletion from the DLI abrogates both graft-*vs-*leukaemia and GvHD effects (Maraninchi *et al*, 1987).

In order to control GvHD, a novel method is being explored whereby the donor lymphocytes are gene modified to express a suicide gene. The general approach involves the *ex vivo* transduction of lymphocytes present within the DLI with a retrovirus encoding the suicide gene (e.g. herpes simplex virus thymidine kinase) (Bonini *et al*, 1997). The DLI is then infused into the patient and, should GvHD develop, the prodrug metabolised by the suicide gene (gancyclovir) is administered to the patient, with the result that the donor T cells are specifically killed thereby controlling toxicity and GvHD (Bonini *et al*, 1997). Unfortunately, the antitumour response is also diminished as a result of the suicide gene system.

## ADOPTIVE T-CELL THERAPY – ‘NATURAL’ T CELLS

Studies focusing on the use of nonspecific modulators of immune activity such as IL-2 have demonstrated that creditable clinical responses can be achieved in certain tumour types, including melanoma and renal cell cancer (Fyfe *et al*, 1995; Atkins *et al*, 1999). These studies have encouraged the development of targeted therapies and, specifically, the clinical testing of antigen-specific T cells for the treatment of melanoma. Antigen-specific T cells isolated directly from tumour biopsies (tumour infiltrating lymphocytes (TILs)) are identified by observing their functional response against melanoma antigens and then subsequently expanded *ex vivo* by IL-2-driven expansion regimes (Dudley *et al*, 2002a). While these results have been extensively reviewed (Rosenberg and Dudley, 2004), it is important to consider the salient points of these studies as a platform to consider gene-modified T cells for future clinical studies. A key observation made from these studies showed that both CD4^+^ and CD8^+^ T cells are required in order to generate an effective response *in vivo* (Dudley *et al*, 2001, 2002b). Furthermore, modulation or elimination of the patient's immune system prior to re-infusion of the expanded T cells was advantageous. A nonmyeloablative preconditioning chemotherapy using cyclophosphamide and fludarabine effectively removed all leukocytes from the patient. The tumour-specific T cells were then re-infused and supported with high-dose IL-2 infusions (Dudley *et al*, 2002b). Interestingly, clinical responses correlated with T-cell persistence *in vivo*, suggesting that survival of the T cells was critical to a successful outcome (Meidenbauer *et al*, 2003). It is not clear whether the beneficial effect of preconditioning was a result of ‘free-space’ created for engraftment through the removal of competing leukocytes or whether the preconditioning had removed important regulatory influences, such as regulatory T cells, thereby allowing the infused T cells to function in a more-friendly immune responsive environment. These studies clearly demonstrated the *in vivo* effectiveness of antigen-specific T cells and also illustrate that manipulating the environment into which the T cells were being re-infused was also critical. However, it is also clear that generating antigen-specific T cells is highly demanding and requires specialised technical expertise, facilities and equipment. This is due to the fact that antigen-specific T cells represent a very small fraction of the total T-cell population. Subsequently, isolating this small number of cells and expanding them to clinically relevant numbers is an issue of significant proportions. Furthermore, many tumour types do not have a significant TIL population or the tumours themselves are not amenable to surgical removal and/or dissection in order to isolate TILs. Consequently, to date, attempts to use TIL therapy have been effectively restricted to trials in renal cell carcinoma and melanoma. In order to address these issues, gene therapy approaches have been explored in order to facilitate the generation of antigen-specific T cells from peripheral blood.

## T CELLS ENGINEERED TO EXPRESS RECOMBINANT TCR GENES

T cells recognise MHC-peptide conjugates on target cells through the paired *α* and *β* chains of the TCR. This pairing confers the antigen specificity of the T cell. One gene therapy approach has involved the molecular cloning of the TCR genes known to be specific for an antigen of choice. These chains are then introduced into T cells usually by means of a retroviral vector. Consequently, expression of the cloned TCR*α* and TCR*β* genes endows the transduced T cell with a functional specificity determined by the pairing of these new genes. In this manner, large numbers of antigen-specific T cells can be generated in a short time period as compared to the longer term culture issues concerning the large-scale expansion of ‘natural T cells’.

There are a number of practical and theoretical issues that are currently being addressed by workers in the field, and recent reviews have provided an in-depth discussion of this specific area (Schumacher, 2002; Willemsen *et al*, 2003; Stauss *et al*, 2004). With relevance to this review, there are three principal issues concerning TCR-based gene therapy, which are of broad relevance to oncologists: isolation and expression of the TCR genes, safety and clinical application.

### Isolation and expression of suitable TCR*α* and TCR*β* chains

The general methodology involves the isolation of T cells that functionally respond to the target antigen from which the TCR chains are cloned using polymerase chain reaction (PCR)-based methods. The resultant DNA products are sequenced to confirm identity and then placed into retroviral vectors suitable for expression in T cells (Clay *et al*, 1999). T-cell receptor chains to date have been generated against known antigens with the principal targeted disease being melanoma due to the fact that MHC restricted antigens are numerous and well defined (Rivoltini *et al*, 2002). Aside from melanoma, there is an increasing diversity of targets being exploited for TCR therapy including MDM-2, a potential target in a wide range of malignancies (Stanislawski *et al*, 2001) and WT-1 targeting in leukaemia (Xue *et al*, 2004). However, the rate-limiting step is the identification and isolation of the critical responding T cell to serve as a source to generate the TCR genes with (generally speaking) a great deal of effort generally required to generate antigen-specific T cells. Development of methodologies that will permit the rapid isolation of TCR genes remains a critical area of development, especially when considering that, for widespread use, TCR genes for each HLA type will be required in order to treat every cancer patient (Murphy *et al*, 2005).

In order to generate a functional T-cell response, both TCR*α* and TCR*β* genes need to be efficiently introduced into the T cell and expressed to levels that will permit sufficient paired complexes to be present on the surface of the transduced T cell. Retroviral gene transfer technologies have been the method of choice since these vectors can efficiently transduce primary T cells (Movassagh *et al*, 2000). Importantly, current retroviral vectors based on the murine leukaemia virus family require the target cell to be undergoing rapid cell cycling to permit efficient transduction (Miller *et al*, 1990). With respect to T cells, cell cycling is stimulated by the activation of T cells using strong mitogenic stimuli (with lectins or antibodies), and this may have a profound effect on the subsequent functionality of the transduced T-cell populations. Other vector systems are being investigated (Chinnasamy *et al*, 2000; Jensen *et al*, 2000); however, these murine retroviral vectors possess a clinical pedigree (Bonini *et al*, 2003) and so remain the principal choice for TCR-based approaches. Expression of multiple TCR genes has been achieved through a number of methods, including transduction with multiple retroviruses encoding single genes (Schaft *et al*, 2003) or single vectors employing internal ribosome entry sites for multiple gene expression (Morgan *et al*, 2003), although further improvements in vector design are required in order to ensure the high-level expression of multiple genes in retroviral vectors.

Critically, primary human T cells transduced with retroviral vectors encoding TCRs functionally respond against tumour cells expressing the target antigen. These responses include cytokine release, proliferation and cytotoxicity (Gao *et al*, 2003; Morgan *et al*, 2003; Schaft *et al*, 2003), indicating that the overall strategy is highly effective in directing the functional activities of T cells. However, the majority of TCRs cloned to date are MHC class I specific and, consequently, direct the functional activity of CD8^+^ T cells. Encouragingly, these MHC class I restricted TCR genes also appear to function in CD4^+^ T cells (Morris *et al*, 2005), suggesting that a polyclonal T-cell response consisting of both CD4 and CD8 T cells is feasible using a single TCR pairing.

### Safety

Tumorigenesis as a result of retroviral insertional mutagenesis has been documented in the case of engineered stem cell therapy for X-linked SCID (Hacein-Bey-Abina *et al*, 2003). However, at present, there are no examples of clinical manifestation of insertional mutagenesis associated with genetic modification of differentiated T cells (Bonini *et al*, 2003), suggesting that the same safety issues which are evident in stem cell therapy approaches are a less significant issue in differentiated T-cell therapy (Stauss *et al*, 2004). However, a specific concern with TCR-based therapy is the fact that the new TCR genes could pair with endogenous TCR genes, with the possible result that T cells with a new and autoimmune specificity could be generated. The probability of this occurring is unknown and the potential danger has to be carefully assessed and balanced against the potential benefit of treating patients with advanced disease. However, more recent studies have focused up engineering the antigen-specific TCRs in order to prevent pairing with endogenous TCR genes (Willemsen *et al*, 2000).

### Clinical application

An important final issue concerning the use of TCR therapy is the fact that dealing with specific TCRs will mean restricting the target patient group to those expressing the correct MHC haplotype, while the reliance upon tumour expression of MHC is central to the therapy. The majority of ongoing studies have focused upon targeting more common MHC haplotypes, however, for widespread application numerous TCR combinations for specific target antigens will have to be developed and validated.

A further complicating factor is that these gene-modified T cells are dependent on the antigen presentation machinery of the tumour. Critically, MHC downregulation is a commonly observed feature of tumours (Garrido *et al*, 1997). In the absence of a suitable MHC-peptide target on the surface of the tumour, T cells expressing tumour-specific TCR genes would be unlikely to respond against the target. Modulation of MHC expression on tumours is possible; however, in the light of these observations, targeting of T cells to recognise intact cell surface protein antigens using antibody-based technologies, thereby avoiding tumour antigen presentation mechanisms, has been the focus of an increasing number of research groups.

## ANTIBODY-TARGETED T CELLS (CHIMERIC IMMUNE RECEPTORS, ENGINEERED T CELLS)

The basis of this field resides in studies performed in the late 1980s, when it was demonstrated that a receptor consisting of a fusion of an antibody domain with the TCR*β* chain could direct T-cell hybridomas to respond against the protein antigen targeted by the antibody. This showed that T cells could respond to antigen independent of TCR–MHC interaction (Gross *et al*, 1989). Given that tumours can frequently lose antigen expression through factors such as the downregulation of MHC expression (Garrido *et al*, 1997), this makes antibody targeted T cells highly attractive. The subsequent 15 years have seen the rapid development of chimeric immune receptor (CIR) technology through to current testing in ongoing phase I clinical trials. Various recent publications have extensively reviewed this area (Hombach *et al*, 2002; Thistlethwaite *et al*, 2005). The aim here is to provide an overview of the current state of antibody targeted T-cell research and to discuss some key scientific areas that are likely to affect upon the immediate translation of this therapy into clinical practice.

**Figure 2:**
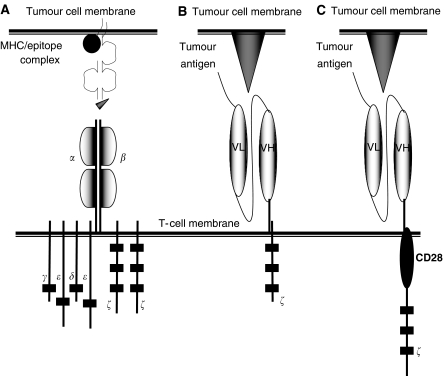
The chimeric immune receptor. (**A**) The T-cell receptor composed of the *α* and *β* chains transmits its signal through the CD3 molecule (*γ*, *ε*, *δ* and *ζ* moieties) following interaction with MHC/epitope complex. This differs from the chimeric immune receptor (**B**) which is composed of an extracellular single chain antibody recognition domain connected to signalling moiety shown in this example as either the CD3 *ζ* molecule (**C**) or as a fusion receptor using the CD28 molecule proximal to the *ζ* moiety. Activation of the chimeric immune receptor can be initiated in the presence of tumour antigen in an MHC independent manner.

T cells using CIRs to target cellular antigens depend on the introduction of a gene encoding the receptor into the T cell. The essential components of a CIR are an antigen targeting domain fused to an intracellular signalling domain anchored to the surface of the T cell by a transmembrane domain (Figure 2). The antigen binding domain most commonly involves a single chain antibody fragment (scFv) consisting of the antigen recognition components of a monoclonal antibody (Gross *et al*, 1989; Hawkins *et al*, 1998), although other protein domains have been successfully used (Mitsuyasu *et al*, 2000). The scFv maintains the specificity of the original antibody, but carries the advantage of small size suitable for expression as part of a CIR. The predominant requisite for the targeted tumour antigen is cell surface expression. Aside from this, the diversity of antigens targeted to date is extensive with the majority of cancer types represented (Table 1).

The signalling domains used have focused on the key signalling molecules CD3*ζ* and the *γ* chain from Fc*ε*RI. A wide number of laboratories have confirmed that CIRs expressed in primary T cells elicit functional responses when cultured with cells expressing the target protein antigen or even purified proteins immobilised on a surface (e.g. on a culture plate) (Haynes *et al*, 2001). These functional responses (including cytotoxicity and cytokine production) are those thought to be important for antitumour activity. A more recent development has involved the engineering of CIRs to incorporate multiple signalling domains. The first receptor of this type involved a fusion of the CD28 receptor with the CD3*ζ* moiety (Finney *et al*, 1998). For full activation of the T cell, multiple signals are required. The TCR/CD3*ζ* complex generates the antigen-specific response of the T cell (namely cytotoxicity and cytokine production). However, in the absence of associated stimuli (so-called costimulatory signals), the T-cell falters during activation and fails to fully respond to antigen (Brocker and Karjalainen, 1995). Costimulatory signals generated through receptors such as CD28 reinforce the TCR/CD3*ζ* signal through further cytokine release (including IL-2) and the upregulation of key antiapoptotic gene expression (Krause *et al*, 1998). Fusion CIRs encoding both CD28 and CD3*ζ* signalling domains (Figure 2C) would be predicted to function more optimally than a CD3*ζ*-only receptor since activatory and costimulatory signals would be generated from the same receptor. Indeed, this appears to be the case, with a growing number of reports confirming that T cells expressing CD28-*ζ* fusion CIRs when stimulated express IL-2 and appear to demonstrate improved antitumour activity *in vitro* and *in vivo* (Eshhar *et al*, 2001; Haynes *et al*, 2002; Maher *et al*, 2002). Driven by the relative success of the CD28-based receptors, other known T-cell costimulatory receptors are being tested as CIRs, with the majority appearing to bring significant improvements to the CD3*ζ* receptor (Finney *et al*, 2004; Imai *et al*, 2004).

Once again, there are certain key issues concerning antibody targeted T cells that are likely to be of primary interest to oncologists. These include antigen selection/selection of targeting moiety, safety and clinical application.

### Antigen selection/selection of targeting moiety

The key attractive feature of this approach is the generic nature of the targeting construct. Since the target is typically a cell surface expressed tumour associated antigen (TAA), the scFv (or other moiety) is not restricted by HLA expression and, subsequently, a single receptor is broadly applicable to all cancer patients as long as the target antigen is expressed on the tumour. Furthermore, with the development of powerful scFv selection technologies (e.g. phage display (Winter *et al*, 1994)), scFv can, at least in theory, be rapidly generated against any protein antigen. However, the selection of the target antigen is of critical importance since the expression of the target antigen on normal tissues could result in toxicity (discussed below).

### Safety

The issues of retroviral insertional mutagenesis apply to antibody targeted T cells as for TCR gene therapy as discussed previously. However, clinical trials are ongoing, where plasmid-mediated gene transfer has been used to avoid some of the issues with retroviral gene transfer. However, this approach is still likely to suffer from the same potential problems of insertional mutagenesis due to integration of the construct into the genome of the T cell.

The issue of target antigen selection is of major importance. The majority of TAAs are antigens, which are overexpressed on tumours. However, expression of these antigens is not restricted to tumour with antigens typically expressed on normal tissues. For example, carcino-embryoinic antigen (CEA) has been extensively targeted in a range of immunotherapy approaches including antibody targeted T cells (Hombach *et al*, 1999; Nolan *et al*, 1999; Gilham *et al*, 2002). CEA is expressed to high levels on tumour, yet is also expressed to far lower levels on normal gastric mucosal surfaces (Hammarstrom, 1999; Francis *et al*, 2002). While there has been no evidence of toxicity in CEA vaccination trials (Berinstein, 2002), clinical trials, which are due to start using antibody targeted T cells, will assess whether gut toxicity is observed as a part of the T-cell therapy. In the absence of truly tumour-specific antigens, any therapy targeting tumour antigens is likely to be associated with a level of toxicity. The severity of this toxicity remains to be established and predicting and managing it will be an important component of any trial protocol. Critically, knowledge of the normal tissue distribution of the targeted antigen and the relative level of expression will be important in order to facilitate this process. In the case of CEA, it is envisaged that the high level of expression on tumour cells coupled with the low levels of expression on a restricted distribution of normal cells will mean that autoimmune toxicities should be minimal.

A final point relates to the use of receptor constructs that encode multiple signalling domains. Ligation of CD28-*ζ* receptor expressing T cells by antigen results in a plethora of signals, which are intended to improve the survival of the engineered T cell. Given that normal cells may express TAAs albeit to low levels, there may be an issue that CD28-*ζ* receptor T cells could be continuously stimulated and therefore produce longer term unwanted side effects. Once again, the potential toxicity will be balanced by the theoretical improved cancer killing, which may be generated by these improved receptor constructs. Until clinical trials have been carried out against a number of target antigens, it is unclear whether these potential toxicities pose a real or theoretical risk.

### Clinical application

Current clinical trials are testing CD3*ζ* and *γ*-based CIRs (Lamers *et al*, 2002) with a trial likely to test CD28-*ζ* receptors in the near future. While testing the function of engineered T cells *in vivo*, these trials are also aiming to investigate whether the general approach to gene modification and culture of T cells is feasible.

## FUTURE DIRECTIONS

Engineered T-cell therapy is in its infancy, although the approach is now being tested in early-phase clinical trials. These clinical investigations are based on observations, which have shown that engineered T cells (either expressing an engineered TCR or antibody receptor) can respond against their desired antigens in a manner that suggests that these T cells may be effective against cancer *in situ*. While highly encouraging, these studies also highlight how little is effectively known about engineered T cells. Issues such as how best to culture and gene-modify T cells are still being addressed. The inclusion of cytokines in addition to IL-2 during culture of the T cells is likely to affect the design of clinical protocols in the near future. Trials with TILs suggest that persistence of T cells is important (Robbins *et al*, 2004), and thus combining the chemotherapy regimes used in TIL trials with engineered T cells may prove to be of major importance. At a molecular level, a basic understanding of how engineered T cells function in response to antigen is also lacking. In addition, the impact of key immune cells that can dampen the immune response (such as regulatory T cells) is not known. There is also a lack of understanding of how gene modification can affect how engineered T cells survive and traffic in the patient's circulation. With these issues in mind, it is likely that combining engineered T cell with preconditioning chemotherapy, IL-2 support and vaccination protocols may all contribute to the *in vivo* effectiveness of this therapy.

## Figures and Tables

**Figure 1:**
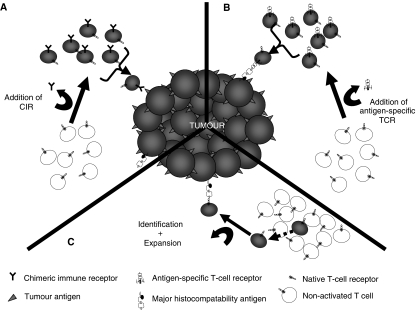
Generation of tumour antigen-specific T cells. Different strategies have been employed to endow T cells with the specificity and power to specifically kill tumour. Large numbers of host T cells can be modified to become tumour reactive by transducing them to express. (**A**) Chimeric immune receptors or (**B**) tumour-specific T-cell receptors using retroviral technology. (**C**) Tumour reactive T cells are identified and grown out of a population of tumour infiltrating lymphocytes. These cells are then expanded for use.

**Table 1:** Viral/tumour associated antigens targeted by chimeric immune receptor T cells

**Targeted antigen**	**Target cells**	**Reference**
CD20	B cell lymphoma	Jensen *et al* (2003)
CD30	Hodgkin's lymphoma	Hombach *et al* (2001)
CEA	Gastrointestinal tumours	Gilham *et al* (2002)
ErbB-2	Breast, ovarian carcinoma	Altenschmidt *et al* (1997)
G250	Renal cell carcinoma	Weijtens *et al* (2000)
Gp120	HIV	Walker *et al* (2000)
MAGE-EA1	Melanoma	Willemsen *et al* (2000)
NCAM	Neuroblastoma	Gilham *et al* (2002)
